# Viral Cell-to-Cell Transmission—Why Less Is More

**DOI:** 10.1371/journal.pbio.1002095

**Published:** 2015-03-17

**Authors:** Lauren A. Richardson

**Affiliations:** Public Library of Science, San Francisco, California, United States of America

## Abstract

During cell-to-cell transmission, only a minute fraction of viral genomes contribute to the progeny. A new study reveals the underlying stochastic processes and explains why these are advantageous for the virus.

Imagine millions of creatures settling on a new planet. How would they repopulate this new world? If they were viruses, they would choose five individuals, at random, to do the job. When viruses infect new cells within a host, they invade that cell with thousands of genomes, but only a small number, on the order of 4 or 5, are successfully replicated (Fig. 1). Why do viruses use this strategy, and how did this arise? A recent study in *PLOS Biology* by Shuhei Miyashita, Masayuki Ishikawa, and colleagues seeks to understand why cell-to-cell (also known as tissue) viral infection involves such a small number of genomes and how this is advantageous to the virus. Fascinatingly, the authors find that genomes are essentially determined at random, but through this inherently random—or stochastic—process, beneficial genomes are selected and defective genomes are expunged.

**Fig 1:**
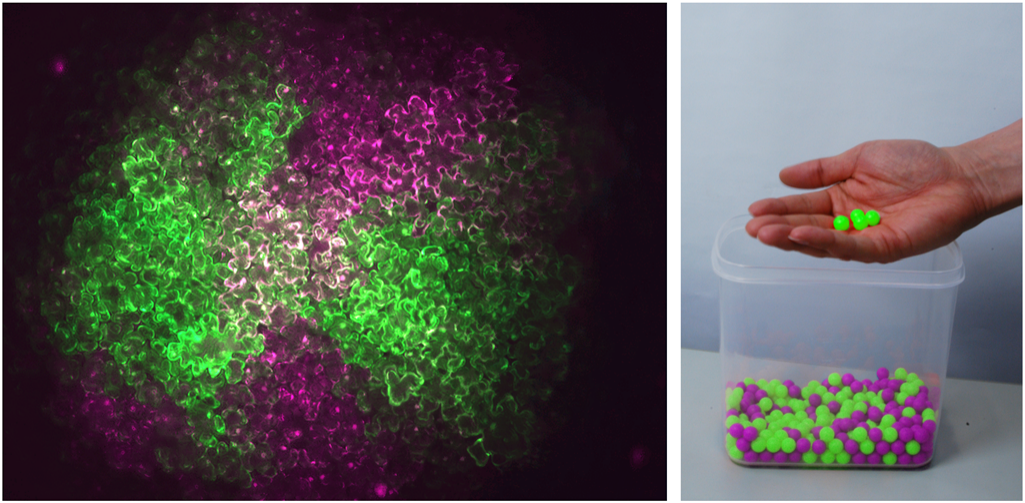
Two differently labeled tomato mosaic virus (ToMV) variants get separated by repeated cell-to-cell transmissions; simple mathematics reveals that every cell infection is established by a small number of viral genomes. Image credit: Shuhei Miyashita.

In this manuscript, Miyashita and colleagues uncover the stochastic nature of viral selection during cell and tissue infection. A process is considered stochastic when it is random and governed by probability, and in biology this usually implies a lack of complex regulatory mechanisms. The authors find that the manner of viral genome selection after cell and tissue infection is completely stochastic and that this characteristic is reflected in nearly every step of the viral life cycle. As mentioned above, thousands of genomes are introduced into a naïve cell after cell-to-cell infection. These genomes are then either degraded by cytosolic nucleases or are translated by ribosomes and sequestered into replication complexes. The genomes that are translated and sequestered are known as the founders. The genomes produced from the founders—the progeny—are released into the cytosol, where they too are either degraded by nucleases or captured and translated by ribosomes. All of these stages—the capture of founders, the number of progeny produced by each founder, and the degradation and replication of the progeny—are stochastic processes, meaning that there is no active regulation on any of these steps. Thus, whether a genome becomes a founder and the amount of progeny that founder produces are based solely on probability. In fact, it is the very stochasticity of these processes that facilitates the selection of more advantageous genotypes.

To investigate the stochastic nature of viral infection and selection, the authors use tagged derivatives of the tomato mosaic virus (ToMV), a positive-strand RNA virus, which they use to infect either tobacco leaves, to model tissue infection, or isolated protoplasts (cells stripped of their cell walls), to model cell infection. Based on results from experiments using two fluorescently labeled ToMV derivatives, the authors develop a simple mathematical model of ToMV single-cell infections, which factors in the stochastic, or random, nature of this process. From this model, they find that, surprisingly, the number of founder genomes is not dictated by a lack of replication sites—the protein complex “factories” needed to replicate the viral genome. The authors use their model to create a simulation of the course of a single-cell infection, which reveals that each founder forms one replication complex, but the number of replication complexes formed by the progeny of each founder is variable and this results in an unequal number of progeny produced from each of the founders. Thus, the ratio between the progenies of the different founder genomes is unequal. The authors call this property SIPA, or stochastic inequality of progeny accumulation.

Additionally, using their model to simulate the infection of 1,000 cells, they find that there is a random variation in the number of founders between different cells, which they call SVFN or stochastic variation in founder number, and this also contributes to the randomness of progeny production. To provide experimental evidence of their model, they use a library of ToMV derivatives in which each copy of the genome includes a unique 10-nucleotide sequence tag. After infection in protoplasts, they amplified and sequenced the tags, allowing them to determine the identity of viral genomes produced in the cell. This experiment showed that the number of founding genomes varied from 2 to 7, with an average of 5, which agreed with their simulation model and with the presence of SVFN. Also, by quantifying the ratio of tags in each infected cell, they saw an unequal production of progeny, demonstrating that SIPA does indeed occur.

Essential to the survival of viruses is the selection on beneficial genomes and removal of deleterious genomes. Mutations in genomes can affect the productivity of a virus in two fundamental ways. They can act “in *cis*,” meaning that their benefits or detriments act exclusively on the genomes in which they lie, or they can act “in *trans*,” in which the beneficial gene products can be shared among the intracellular viral population. To determine if the stochastic processes—the variation in the number of founders (SVFN) and the unequal production in the progeny (SIPA)—lead to increased selection on *trans*-acting factors, the authors developed a second simulation: one that models evolution. This simulation compares the rate of selection on advantageous genomes between cell infection simulations that undergo the stochastic processes SVFN and SIPA versus those that do not. By beginning with the assumption that 20% of the genomes are advantageous and 80% are defective, their model shows that after only four rounds of successive infection cycles (akin to an infection passing through four cells) the population of advantageous genomes increased from 20% to 90% when SVFN and SIPA were factored in—significantly faster than when these two stochastic processes were not taken into account.

To determine whether stochastic processes also aided in the selection of *cis*-acting factors, the authors infected cells with a ToMV variant containing a mutation that causes a reduced ability to synthesize genomic RNA. Inoculating this mutant ToMV in equal parts with normal ToMV, they see, as predicted by their model, that normal ToMV accumulated at a higher rate and suppressed the production of the mutated ToMV. The authors named this property EBPA, or enhanced bias in progeny accumulation.

The selection of *cis-* and *trans-* acting factors, which require stochastic events both within a cell after infection and between cells during tissue infection, results in a handful of randomly selected viral genomes that will accumulate in each cell in preparation for transmission to a new host. Since deleterious mutations will arise in every cell infection cycle, the stochastic processes result in a suppressed proportion of defective genomes within a host. Thus, cell infection and cell-to-cell transmission act as a stringent winnowing process to select for the most beneficial genomes to produce for host-to-host transmission—the most inefficient and challenging step in the viral life cycle.
